# Attenuation of Zn-induced hyperleptinemia/leptin resistance in Wistar rat after feeding modified poultry egg

**DOI:** 10.1186/1743-7075-9-85

**Published:** 2012-09-19

**Authors:** Satish Kumar Taneja, Reshu Mandal, Aman Chechi

**Affiliations:** 1 Department of Zoology, Panjab University, Chandigarh 160014, India

**Keywords:** Zn, Cu, Mg, Hyperleptinemia, Leptin resistance, Modified poultry egg, Conventional egg

## Abstract

**Background:**

The prevalence of obesity is increasing exponentially world over. Leptin resistance/hyperleptinemia is attributed to its cause in majority of the obese humans where mutation in genetic component or ob gene has not been found operative. The generation of oxidative stress was suggested as its cause. In our previous study, we have reported that the inclusion of antioxidant enriched modified poultry egg (ME) in diet reversed the ionic imbalance and ameliorated the oxidative stress caused by excessive Zn in diet. In the present study, the efficacy of ME verses conventional egg (CE) was tested on Zn-induced leptin resistance in rat model to ascertain if the supplementation of antioxidants in the form of egg can reverse Zn-induced leptin resistance to leptin sensitive state.

**Methods:**

Hyperleptinemia was induced in rats by feeding them Zn-supplemented hyperleptinemic diets-I and II (Zn-HL-Diet) for 2 months. Thereafter, half of them were fed either on CE or ME mixed Zn-HL-diets I and II for another two months. The data was analyzed applying one way Anova and Tukey’s HSD post hoc test.

**Results:**

The results revealed that food intake, gain in body weight, height and number/unit surface area of intestinal microvillus and serum leptin, glucose, insulin and cortisol were higher in CE and Zn-HL-Diet treated groups; serum Zn, Cu, Mg were higher and Cu and Mg in tissues were lower in them than the control group. In ME treated groups, these parameters were lower and were close to the control group. These changes resulted from the restoration of ionic balance of Zn, Cu and Mg in the blood serum and tissues including liver and hair in ME treated rats.

**Conclusion:**

The data suggest that Zn-induced leptin resistance can be attenuated through restoring the ionic balance of Zn, Cu and Mg through inclusion of antioxidants in diet such as these modified eggs. But further clinical studies are required before they are put to use for human consumption.

## Background

Obesity is one of the most serious physiological disorders as it increases the risks of hypertension, non-insulin dependent diabetes mellitus, coronary artery disease, stroke, renal failure, gall bladder stones and certain types of cancer. Previous study on the molecular defect underlying the development of obesity in genetically recessive ob/ob mouse led to the discovery of leptin, a cytokine-like factor, produced predominantly in white adipose tissue [1]. Leptin administration in *ob/ob* obese mice causes an increase in energy expenditure and decrease in appetite [2-5] by depressing hypothalamic levels of orexigenic neurotransmitters [5,6]. It led to the assumption that human obesity may also be a leptin-deficient state that could be treated with exogenous leptin administration. However several population studies have failed to demonstrate such mutations [7-9] (except in a small fraction of population), instead majority of them have increased leptin levels, indicating that obesity is a leptin resistant states in most obese [9].

The leptin gene expression is mainly influenced by food intake, greatly reduced after fasting, increased by re-feeding both in rodents and humans correlated with changes in insulin and cortisol [10,11]. In our previous study, we have reported that excessive Zn in diet induces obesity, hyperinsulinemia and hypercortisolemia as a consequence of ionic imbalance of Zn, Cu and Mg [12] resulting in oxidative stress [13] and leptin resistance in Wistar rats [14].

The implication of these reports has a direct relationship of excessive Zn in diet to the obesity and obesity related diseases, the incidence of which are increasing consistently during the past two decades due to its indiscriminate use in agriculture and animal husbandry practices and its inclusion in baby foods that has led to its increased consumption [15,16]. This warrants some mechanism which may not allow creating the ionic imbalance due to excessive consumption of Zn in diet and preventing the onset of oxidative stress, leptin resistance and protect the consumers from obesity and obesity related diseases. Aiming this, the poultry egg is modified with lower level of cholesterol, enriched with Cu, Mg, vitamin E and linolenic acid (Indian Patent No. 253740). The severity of physiological disorders due to oxidative stress caused by excessive Zn decreased significantly on feeding these modified poultry egg (ME) mixed diets by restoring the minerals levels in blood serum and tissues as reported in the previous study [17]. Therefore, the present work was undertaken to study the effect of ME verses conventional egg (CE) on Zn induced hyperleptinemia [14] and results are reported in this paper.

## Methods

For the present investigations, 42 male Wistar rats were procured from the Central Animal House (Panjab University, Chandigarh) and maintained in plastic cages with stainless steel top grills at room temperature 25-28°C with 12:12 hours L:D cycle and 70-80% relative humidity. They were used in this study after taking approval of the protocol from Institutional Ethics Committee. ^$^Semi-synthetic basal diet was employed with increasing Zn concentration to induce hyperleptinemia [14].

^$^The semi**-**synthetic basal diet contained [12] (g/100 of diet) : Casein, 30; Agar, 2.0; Corn oil, 5; Cellulose, 8; Sucrose, 51; Vitamin mixture 0.5 [Vitamin mixture (mg/Kg): Ascorbic acid, 500; Biotin, 4; Calcium pentothenate, 320; Choline chloride, 2500; Folic acid,10; Inositol,1000; Nicotinic acid, 300; Pyridoxine HCl, 180; Riboflavin, 120; Thiamin HCl 200; α-tocopherol acetate (E), 60; Cyanocobalamin,0.40; Retinol, 0.30; Ergacalciferol, 0.0031] and mineral mixture,3.5 [Mineral mixture (gm/Kg) : CaH_2_P0_4_, 25.30; C0Cl_3_, 0.04; CuCl_2_, 0.10; FeS0_4_.7 H_2_0, 0.60; MnS0_4_.H_2_0, 0.30; MgS0_4_.H_2_0, 4.05; KCl, 3.43; KI, 0.004; Na_2_C0_3_, 1.15; NaF,0.008; ZnS0_4_.7 H_2_0, 0.088g].

### Modification of basal diet

The basal diet was divided into 3 parts and modified as (i) Basal diet containing 20 mg Zn/Kg diet for control group-I, (ii) Zn- induced-hyperleptinemic-diet-1 (Zn-HL-diet-I) containing 40 mg Zn/kg diet and (iii) Zn induced hyperleptinemic-diet II (Zn-HL-diet-II) containing 80 mg Zn/Kg diet by accordingly increasing ZnS0_4_.7 H_2_0 in the basal diet.

For the preparation of these diets, the ingredients were weighed. The water soluble vitamins were thoroughly grounded and mixed along with sucrose, while the fat soluble vitamins were dissolved in corn oil. Agar, used as a binding agent was dissolved in 100 ml of double distilled deionised water with constant stirring on water bath maintained at 60°C. On its cooling to 40°C, the contents of each diet were thoroughly mixed in separate containers. The dough so formed was put in Petri dishes and solidified in refrigerator for 3 hours to harden the wet fresh diets. The solidified diets were then cut into small pieces of 2 cm × 2 cm × 2 cm size and stored in the containers at < −4°C.

In order to study the effect of ME, test diets-A and B were prepared after adding four liquid ME (50 g per egg/kg of diet) in Zn-HL-diet-I (Diet-A) and Zn-HL-diet-II (Diet-B). Similarly, the test diets C and D were prepared after adding 4 liquid CE (50 g/kg diet) in Zn-HL-diet I (Diet-C) and Zn-HL-diet II (Diet-D) respectively.

### Experimental design and feeding of rats

The male Wistar rats (n = 42) were divided into three groups: control group-I (control, n = 6), group-II (n = 18) and group-III (n = 18) and were fed on basal diet, Zn-HL-diet-I and Zn-HL-diet-II respectively for 60 days. On day 60, the rats of Group-II were subdivided equally into 3 subgroups as subgroup-II, subgroup-IICE, subgroup-IIME, and similarly group-III into subgroup-III, subgroup-IIICE and subgroup-IIIME including 6 rats in each subgroup. The control group-I, subgroup-II (group-II) and subgroup-III (group-III) were continued to feed on their respective basal diets, Zn–HL-diet-I and II for another 60 days completing 120 days. The rats of subgroups-IIME and IIIME were shifted to respective ME-mixed Zn-HL-diet-I (diets-A) and II (diet-B), and the rats in subgroups-IICE and IIICE on CE-mixed Zn-HL-diet-I (diet-C) and II (diet-D) respectively for another 60 days thus completing 120 days of the experiment. The data of food intake and body weight were recorded on monthly basis during dietary treatment. They were sacrificed using diethyl ether as anesthesia at the end of the experiment.

### Hormones and mineral analysis

The blood was collected into metal free centrifuge tube by puncturing the heart of rats in each group and subgroups after 120 days of treatment and the blood serum was separated and poured into separate metal free plastic vials. The haemolysed blood samples were excluded in the present study. Serum glucose was estimated using commercially available diagnostic kit (Reckon Diagnostics PVT, LTD, Baroda, India). Hormonal analysis included insulin [18,19] by a solid phase two site enzyme immunoassay (DRG Diagnostics, USA), cortisol [20,21] by enzyme immunoassay (Microtiter strip kit supplied by Immune-Biological Laboratories, Hamburg) and leptin [22,23] by solid phase two site enzyme-immunoassay (Mediagnost Aspenhaustr, D-72770 Reutlingen/Germany).

The minerals including Cu, Zn and Mg were estimated in serum, liver and hair. The tissues were digested in nitric acid and perchloric acid mixture (3:1 v/v) on a sand bath until a white ash was formed. The ash so obtained was dissolved in 6 ml of 10 mM HN0_3_ in triplicates and filtered through ash free filter paper before analysis. Minerals Cu, Zn and Mg were then estimated on atomic absorption spectrophotometer (Electronic Corporation of India Limited Hyderabad- AAS 4139) using hollow cathode lamps (213.9, 324.8 and 285.2 nm for Zn, Cu and Mg respectively). Standards (sigma) for these metals were prepared by dilution in triple distilled deionized water.

### Pathology of intestinal epithelium

Pathology of intestinal epithelium was studied on transmission electron microscope (TEM). For this study, procedure described by **collect**[24] was followed. Small pieces (1 mm cubic) of small intestine (10 cm above the caecum) were taken from freshly killed rats and washed in 0.2 M phosphate buffer and fixed in modified **karnovsky’s** fixative (2.5% **glutaraldehyde**, paraformaldehyde, 0.2 M phosphate buffer). Post fixation was carried out in 1% osmium tetra oxide (1% OsO_4_ diluted in 0.2 M phosphate buffer, pH 7.2) for 1hour at 4°C. The intestinal pieces were dehydrated in ascending grades of acetone (30-100%) and cleared in toluene. They were infiltrated with **spurr’s** low viscosity embedding medium by passing them through a sequence of solutions until the dehydrating agent was completely replaced by the final embedding mixture. The sections were cut on RMC-MTX ultra microtome in the range of 600-800Aº and stained with uranyl acetate followed by lead citrate to increase the contrast.

### Analysis of eggs

The MEs were obtained from the hens after feeding them on modified bird’s feed as per specification given in Indian patent no. 253740. The CEs were obtained from the Bee jee poultry farm, Panchkula, India. They were analyzed for cholesterol [25,26], triglycerides [27], total lipids [28], proteins [29], carbohydrates [30,31], vitamin E [32,33], linolenic acid and minerals (Zn, Cu and Mg) as described previously [17].

### Statistical analysis

The data were subjected to statistical analysis applying one way ANOVA and Tukey’s post hoc test (Honestly significant difference).

## Results

### Composition of ME versus CE

ME and CE typically (50 g liquid/egg) contained total lipid, 5.84 ± 0.43 versus 7.8 ± 0.07 (g/egg, 25.13% less); cholesterol, 110.77 ± 3.19 versus 205.92 ± 6.02 (mg/egg, 46.21% less); triglycerides, 1.37 ± 0.01 versus 5.5 ± 0.06 (g/egg, 75.1% less); protein, 6.5 ± 0.12 versus 6.7 ± 0.14 (g/egg, 2.98% less); carbohydrate, 0.63 ± 0.02 versus 0.69 ± 0.15 (g/egg, 8.69% less); vitamins E, 10.35 ± 0.32 versus 3.22 ± 0.03 (mg/egg, 68.89% higher); linolenic acid, 73.84 ± 3.57 versus 36.0 ± 0.01 (mg/egg, 51.24% higher); Cu, 3.02 ± 0.04 versus 1.36 ± 0.03 (mg/egg, 54.97% higher); Mg, 2.02 ± 0.01 versus 0.61 ± 0.01(mg/egg, 69.80% higher) and Zn, 0.65 ± 0.01 versus 2.98 ± 0.05 (mg/egg, 78.19% less).

### Food intake and body weight

**Figure 1:**
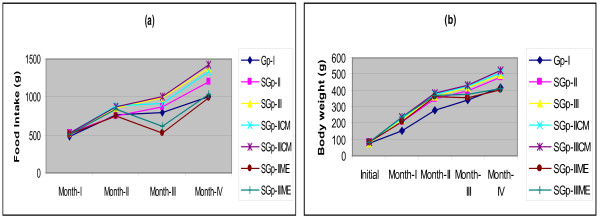
**Line graph showing mean month wise food intake (a) and body weights (b) of Wistar rats in groups control group-I, subgroup-II, III, IICE, IIICE and IIME and IIIME during the 4 months of dietary treatment. **[Values are mean ± SEM of n= 6].

The food intake and gain in body weight (g) showed significant variation on monthly basis. They were higher in rats of group-II (subgroup-II) and group-III (subgroup-III). The rats in subgroup-IICE and subgroup-IIICE fed on diets containing CE on 60th day and onwards continued to consume more food and gained more body weight (Figure 1a, 1b). There was no significant variation between subgroup-IICE and subgroup-IIICE but the food intake was higher by 24.46% in subgroup-IICE and 29.49% in subgroup-IIICE than control group-I and body weights were higher by 17.7% in subgroup-IICE and 19% in subgroup-IIICE as evaluated on day 120 of the experiment. In contrast to this, the food intake and gain in body weight in rats of subgroup-IIME and subgroup-IIIME fed on diets containing ME on day 60 started gradually declining compared to subgroup-II and subgroup-IICE, and subgroup-III and subgroup-IIICE and approached closer to the control group-I on day 120. The food intake was 25.54% and 27.83% (Figure 1a) and their body weight were 20.45% and 21.15% (Figure 1b) higher in subgroup-IICE and IIICE respectively than the subgroup-IIME and subgroup-IIIME at the end of the experiment.

### Serum leptin, glucose, insulin and cortisol

**Figure 2:**
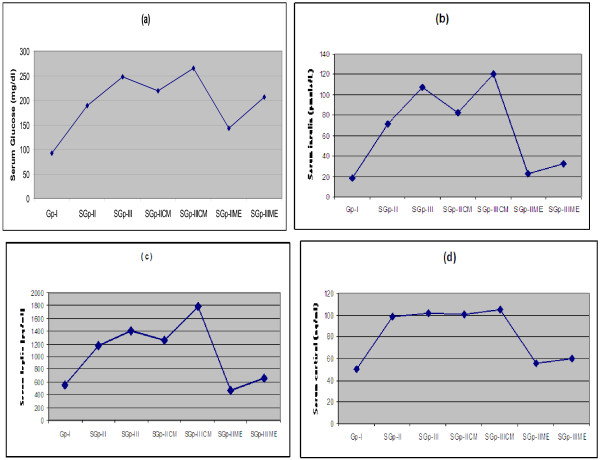
**Line graphs of blood profile showing serum glucose (a), insulin (b), leptin (c) and cortisol (d) in male Wistar rats in control group-I, subgroup-II, III, IICE, IIICE and IIME and IIIME after the 4 months of dietary treatment. **[Values are mean ± SEM of n= 6].

Along with the food intake and gain in body weight, the serum leptin (pg/ml) was found to increase in subgroup-II and subgroup-IICE, and subgroup-III and subgroup-IIICE (Figure 2c). Its levels were 62.90% and 63.61% higher in subgroup-IICE and subgroup-IIICE than control group-I. Its levels in subgroup-IIME and subgroup-IIIME did not show any significant difference in comparison to the control group-I suggesting that the ME did not allow the leptin level to increase even if the diets contained higher Zn level in diets similar to subgroup-II and subgroup-III and subgroup-IICE and subgroup-IIICE. Coinciding with the food intake, body weight, serum leptin concentrations, the serum glucose (mg/dl), insulin (pmole/L) and cortisol (ng/ml) concentrations increased in group-II, subgroup-IICE and subgroup-III, subgroup-IIICE than the control group-I suggesting that these rats developed hyperinsulinemia and hypercortisolemia (Figure 2a, 2b, 2c, 2d). In contrast to this, serum glucose, insulin and cortisol levels in subgroup-IIME and subgroup-IIIME were closer to the control group-I. The glucose, insulin and cortisol levels were 34.88%, 72% and 44.6% in subgroup-IIME and 22.14%, 72.5% and 42.9% (Figure 2a, 2b, 2c, 2d) in subgroup-IIIME less than the respective CE treated groups suggesting that the MEs did not permit the rise of serum glucose, insulin and cortisol in spite of the level of Zn in their diet comparable to diets fed to the rats in subgroup-II, IICE and subgroup-III and IIICE.

### Intestinal pathology

**Figure 3:**
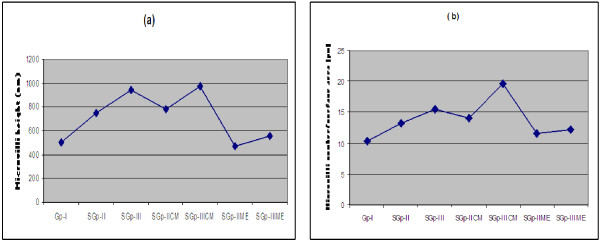
Line graphs showing mean height (a) and number/unit surface area (b) of microvilli of mucosal epithelial cells of intestine in male Wistar rats in control group-I, subgroup-II, III, IICE, IIICE and IIME and IIIME after 4 months of dietary treatment.

The TEM studies of intestinal segment (Additional file 1) revealed a significant increase in the absorptive surface area reflected as an increase in mean height (nm) and number/unit surface (μm) area of microvilli of its mucosal epithelial cells in subgroup-II**CE** (Additional file 1b) and subgroup-III**CE** (Additional file 1c) than that of control group-I (Additional file 1a). The mean height was found to be less by 39.9% in subgroup-IIME and 43.1% in subgroup-IIIME whereas the microvilli number was less by 17.6% in subgroup-IIME (Additional file 1d) and 38.3% in subgroup-IIIME (Additional file 1e) than those of their respective CE treated groups and were very close to the rats in control group-I (Figure 3a, 3b). The increase in height and number/unit surface area in subgroup-II and IICE, and subgroup-III and IIICE were suggestive of the elevated capability of the intestine to absorb the nutrients per unit time in CE treated rats than those of ME treated rats or control group. [Electro-micrographs of intestinal mucosal epithelial cells of subgroup-II and subgroup-III are not shown because the changes in their cytoarchitecture were similar to subgroups- IICE and IIICE].

### Minerals

**Figure 4:**
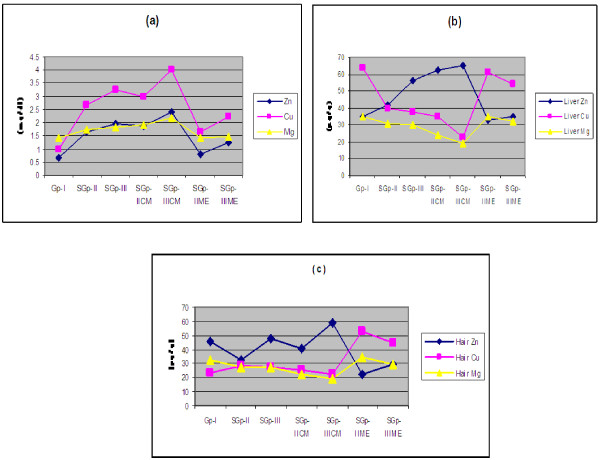
**Line graphs showing mean serum (a), liver (b) and hair (c) Zn, Cu and Mg concentrations in male Wistar rats in control group-I, subgroup-II, III, IICE, IIICE and IIME and IIIME after 4 months of dietary treatment.** [Values are mean ± SEM of n= 6].

Serum Zn, Cu and Mg (mg/dl) concentrations increased while the tissue (liver and hair) Cu and Mg concentrations (μg/g) decreased significantly in group-II, IICE and group-III, IIICE compared to that of control group-I (Figure 4a, 4b, 4c). The tissue concentrations of Zn were significantly less in sub-group-IIME and sub-group-IIIME than the CE treated rats and were close to control group-I (Figure 4b,4c).

## Discussion

The data of food intake and body weight revealed that during the first two months of the experiment, when no egg was added in Zn-HL-I and II diets of group-II and III, the gain in body weight and food intake increased with increase in Zn concentrations in their diets than the control group-I. On addition of eggs in their diets after two months of feeding on this diet, the food intake and gain in body weight continued to display consistent rise which were more in subgroup-IIICE than subgroup-IICE where as their rise in subgroup-IIME and subgroup-IIIME were less than CE treated groups but were higher than the control group-I after the completion of 3 months and approached closer to control group-I at the end of the 4^th^ month of the dietary treatment. This suggested that addition of CE in Zn-HL-diets did not affect the body mass promoting factors. On the contrary, the ME resulted a reduction in food intake and slower gain in body weight than the CE treated rats and were comparable to control group-I suggesting its positive role in regulation of food intake and body weight in a manner similar to the control group-I.

### The ultra structure of intestinal segment

The increase in absorptive cell surface area both in terms of height and number per unit surface area of intestinal microvillus in group-II, subgroup-IICE and subgroup-III, IIICE than IIME and IIIME (Additional file 1) implied that the former two CE treated rats or Zn-HL-diet fed rats have the capability of absorption of more nutrients than the ME treated rats or control group rats as the absorption rate at intestinal level is dependent on availability of its surface area. The greater surface area transfers more molecules from the intestinal lumen to the epithelial cells and to the blood stream at a faster rate than those which have lesser surface area. This change in cyto-architecture of the intestine in these rats possibly imposed two effects: firstly it resulted in absorption of more nutrients from the diet and secondly caused evacuation of stomach faster by relieving the effect of inhibitors on the stomach motility. These two effects were manifested in terms of higher consumption of food and more gain in body weight in Zn-HL-diet fed and CE treated rats in comparison to the ME treated rats.

However, both food intake and body fat mass had been reported to be regulated by leptin [3,34,35]. In the present study, the serum leptin level was evaluated higher in Zn-HL-diet and CE treated rats than those of ME treated rats suggesting that the addition of ME in Zn-HL-diets resulted in a significant reduction of circulating leptin which was less than that of either Zn supplement and CE treated rats. Its level in subgroup-IIME rats was equal to control group-I while in that of subgroup-IIIME, it was approximately 23% higher than the control group-I.

Leptin primarily secreted by white adipocytes, targets receptors in neurons of hypothalamic nuclei for the central expression, and signaling of orexigenic and anorexigenic neuropeptides [36]. The administration of leptin in recessive ob/ob mice lacking leptin gene, results in the reduction in food intake and body fat mass in them. However in subgroups-II (group-II), IICE and subgroups-III (group-III), IIICE, the higher level of leptin could not proportionally reduce either food intake or caused reduction in body fat mass suggesting that leptin failed to inhibit the orexigenic neuropeptide signaling to an extent that may be equal to control group-I. In fact, its higher levels in groups ***per se*** caused opposite effect in them which suggested its resistance to orexigenic receptors. Leptin resistance appears when threshold level of serum leptin above which its increase does not translate into proportional increase in cerebrospinal fluid or brain leptin level [37]. This in turn may result in apparent leptin resistance leading to increase in food intake and higher gain in body weight as observed in subgroups-II and III also reported previously [14], and subgroups-IICE and IIICE respectively in the present study. In contrast, the leptin level in subgroup-IIME and IIIME were evaluated significantly less than those of Zn-HL-diet and CE mixed diet fed groups suggesting that the ME in Zn rich diet did not allow developing leptin resistance as manifested in term of lesser food intake and lesser gain in body weight. Their food intake and gain in body weight, and leptin levels were almost close to the control group at the end of the experiment.

The increased absorption rate of nutrients due to increased surface area of absorptive mucosal epithelial cell of intestine in subgroups-II, IICE and III, IIICE seemed to be the possible reason for hyperleptinemia in them since excessive absorption of nutrients stimulated the release of excessive insulin from β cell of pancreas as recorded in subgroups-II, IICE and III, IIICE for the transport of extra available nutrient to the fat cells for maintaining the homeostatic state of blood glucose leading to the growth of fat cell. This lends further support to the reports that prolonged insulin infusion or supra-physiological insulin level markedly increases leptin level [38-41]. This further elucidated in this study when the absorptive mucosal epithelial cell of the subgroups-IIME and IIIME were taken into account. The absorptive surface of intestine was equal in subgroup-IIME and control group-I so was the leptin. In subgroup-IIIME, it was more than control group-I and so was higher leptin level. This was further supported by data of insulin evaluated in this experiment where its level increased with increase in Zn concentration in diets of subgroups-II, IICE and III, IIICE and were significantly lower in subgroups-IIME and IIIME comparable to control group-I. This suggested that serum leptin level was not regulated by Zn directly but through increase or decrease of absorption rate of nutrients which influenced the circulating insulin levels.

The higher insulin production increases C-reactive protein in the blood which stimulates the corticotrophin releasing hormone [42-44]. Leptin is positively correlated to the corticotrophin releasing hormone since its level has been seen to decrease with its reduction [45]. Higher levels of Zn-induced glucocorticoid cortisol observed in subgroups-II, IICE, III and IIICE supported the concept that the increased synthesis of corticotrophin releasing hormone resulted in hyperleptinemia. Hypercortisolemia has been reported to interfere further with leptin’s interaction with its receptor resulting in central leptin resistance in them [45,46]. This was manifested in this study by the fact that the Zn-HL-diet and CE mixed diets fed groups of rats continued to feed more and gained more body weight in spite of their higher circulating leptin levels. Active leptin controls the food intake and fat mass of the animals but it failed to do so in these rats due to the onset of leptin resistance. In ME treated rats, there was a lesser degree of adiposity and lesser leptin production and its secretion from adipose tissues in the circulating blood than those of subgroups-II, IICE, III and IIICE.

The changes in leptin levels in different groups of rats in this study can be attributed to the changes in the mineral status of Zn and Cu. Zn levels were higher in liver, hair and blood serum, and Cu levels were significantly less in liver and hair but higher in blood serum in subgroups-II, IICE and III, IIICE than the control group-I. The change in leptin level in ME treated rats linked to the level of Cu in it, which resulted in the reduction of Zn and rise of Cu in their tissues (hair and liver) in subgroups-IIME and IIIME than those of subgroups-II and III as well as subgroups-IICE and IIICE respectively (Figure 4a, 4b, 4c). The rise in Cu and fall of Zn in tissues resulted in the fall of leptin close to control group in subgroups-IIME and IIIME leading to normoleptinemia both in its synthesis and activity in them. Accordingly, it resulted in the reduction of food intake and consequently less absorption of nutrients and resultant reduction in gain in body weight recorded in subgroups-IIME and IIIME rats. The dietary Cu supplementation has also been reported to reduce fat depth [47] by not affecting the leptin gene expression but through lypolytic activity of Cu in adipose tissue [48]. The reduction of fat mass caused by Cu in turn contributed to the reduction in serum leptin level [49].

Further the levels of Zn, Cu and Mg that have been found to alter both in serum and tissues of subgroups-II, IICE, III, IIICE compared to the control indicating that Zn when in excess even in pharmacological doses in diet, over a period of time, replaces Cu and Mg in tissues leading to their leaching into the blood and excretion in urine resulting in their deficiencies. This is because of hypercortisolemia which stimulates the synthesis of Cu containing protein ceruloplasmin and Zn binding protein metallothionein in liver and other tissues. Once ceruloplasmin is synthesized, it is transported into the blood resulting in a reduction of Cu in tissues with concomitant increase in serum Cu and stimulated increase of metallothionein elevates Zn concentration in tissues. This leads to the imbalance of Cu and Zn concentrations in the serum and different tissues [17]. The deficiency of Cu in soft tissues have been shown to decrease Cu-metalloenzymes notably Zn-Cu superoxide dismutase, catalase and selinoglutathione peroxidase which form active component of antioxidant defense system [50,51]. Their reduction results in proinflammatory action by increasing C-reactive protein which is positively correlated with interleukin that induces production of C-reactive protein resulting in hypercotisolemia [52], a contributing factor in inducing hyperleptinemia as observed in subgroups-II, IICE and III, IIICE. Whereas, ME treatment to subgroups-IIME and IIIME rats, resulted in reversal of Zn, Cu and Mg levels in soft tissue approaching close to control group relieving the oxidative stress by restoring antioxidant enzyme activities [50] and alteration in the pathways linked to increased oxidative stress and free radical formation leading to normocortisolemia. Thus the reversal of ionic balance, intestinal architecture, serum insulin and cortisol collectively contributed to normoleptinemia in rats fed ME.

## Conclusion

The data **suggest** that Zn-induced leptin resistance due to increased oxidative stress can be attenuated through restoring the ionic balance of Zn, Cu and Mg through inclusion of antioxidants in diet such as found in these modified eggs. But further clinical studies are required before they are put to use for human consumption.

## Competing interests

The authors declare that they have no competing interests.

## Authors’ contributions

Regarding the contribution of each author, the first author (SKT, corresponding author) has supervised and coordinated the present research work. Other two authors (RM and AC) have carried out the transmission electron microscopy and biochemical analysis. The drafting and editing of manuscript was carried out by SKT and RM. All authors read and approved the final manuscript.

## Supplementary Material

Additional file 1 (a-e) Electro-micrographs of intestinal mucosal epithelial cells (jejunum) showing the microvilli (MV), terminal web (TW), nucleus (N), mitochondria (M) and endoplasmic reticulum (ER) in control group-I (a), subgroup-IICE (b), subgroup-IIICE (c), subgroup-IIME (d) and subgroup-IIIME (e) respectively (subgroup-II and subgroup-III, not shown because the changes in their cytoarchitecture were similar to subgroups- IICE and IIICE). Click here for file
