# Supplementary material for: Attenuation of Zn-induced hyperleptinemia/leptin resistance in Wistar rat after feeding modified poultry egg
Source: Nutr Metab (Lond). 2012 Sep 19;9:85. doi: 10.1186/1743-7075-9-85 (PMC3514344; doi:10.1186/1743-7075-9-85)

**Additional 1**.  (a-e)  Electro-micrographs of intestinal mucosal epithelial cells (jejunum) showing the microvilli (MV), terminal web (TW), nucleus (N), mitochondria (M) and endoplasmic reticulum (ER) in control group-I (a), subgroup-IICE (b), subgroup-IIICE (c), subgroup-IIME (d) and subgroup-IIIME (e) respectively (subgroup-II and subgroup-III, not shown because the changes in their cytoarchitecture were similar to subgroups- IICE and IIICE)


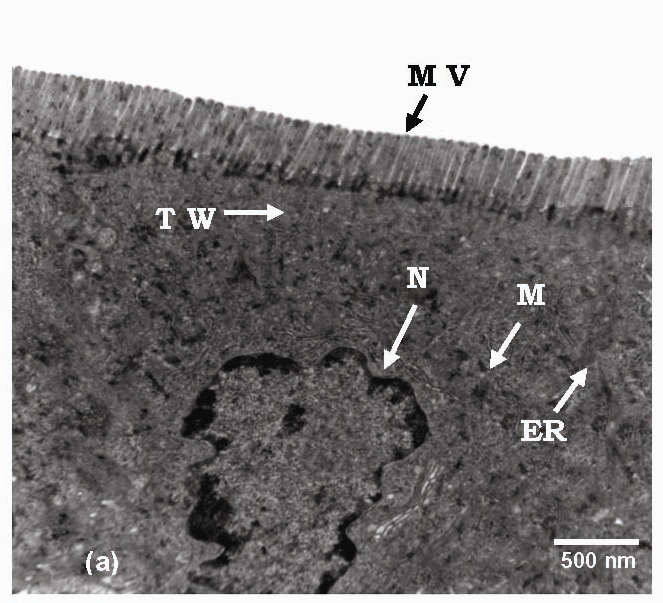


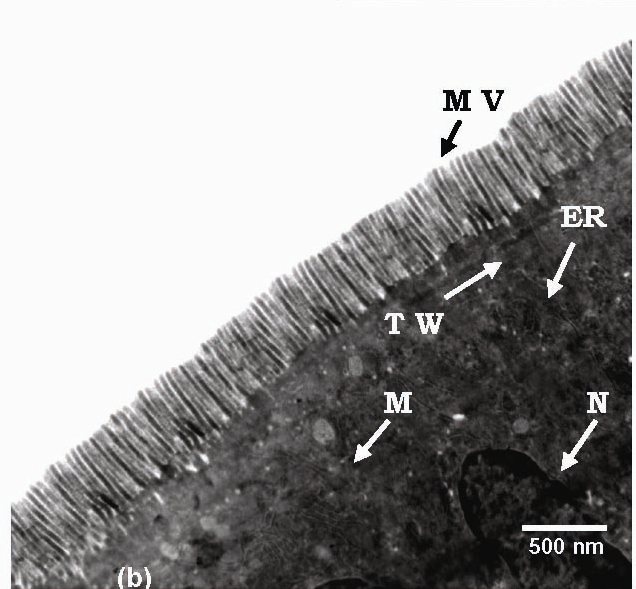


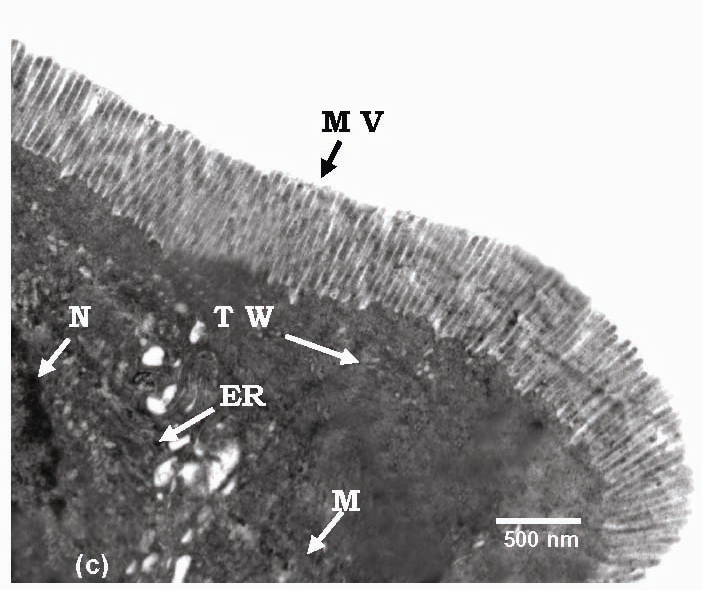


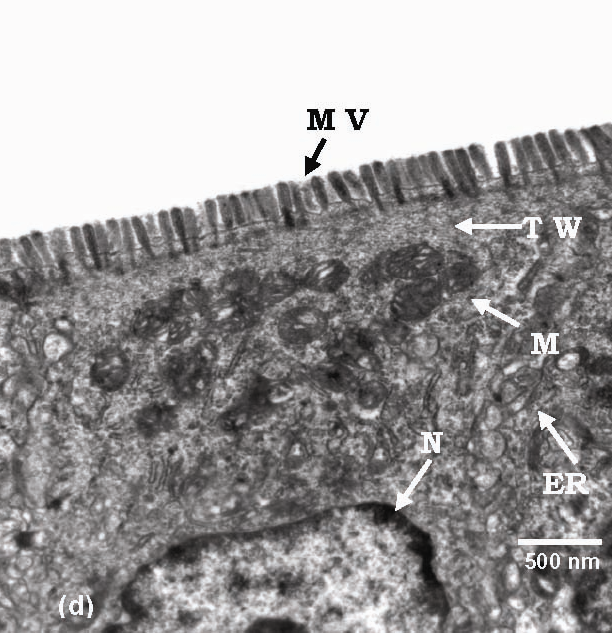


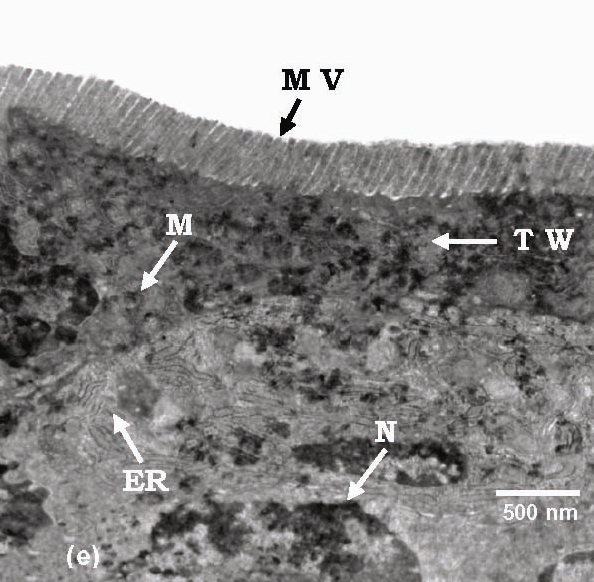

Supplement: Additional file 1 — (a-e) Electro-micrographs of intestinal mucosal epithelial cells (jejunum) showing the microvilli (MV), terminal web (TW), nucleus (N), mitochondria (M) and endoplasmic reticulum (ER) in control group-I (a), subgroup-IICE (b), subgroup-IIICE (c), subgroup-IIME (d) and subgroup-IIIME (e) respectively (subgroup-II and subgroup-III, not shown because the changes in their cytoarchitecture were similar to subgroups- IICE and IIICE). [file 1743-7075-9-85-S1.doc]
